# Structure and sequence at an RNA template 5′ end influence insertion of transgenes by an R2 retrotransposon protein

**DOI:** 10.1261/rna.080031.124

**Published:** 2024-09

**Authors:** Sarah M. Palm, Connor A. Horton, Xiaozhu Zhang, Kathleen Collins

**Affiliations:** Department of Molecular and Cell Biology, University of California at Berkeley, Berkeley, California 94720, USA

**Keywords:** R2 retrotransposon, non-LTR retrotransposon, HDV-like ribozyme, transgene, genome engineering

## Abstract

R2 non-long terminal repeat retrotransposons insert site-specifically into ribosomal RNA genes (rDNA) in a broad range of multicellular eukaryotes. R2-encoded proteins can be leveraged to mediate transgene insertion at 28S rDNA loci in cultured human cells. This strategy, precise RNA-mediated insertion of transgenes (PRINT), relies on the codelivery of an mRNA encoding R2 protein and an RNA template encoding a transgene cassette of choice. Here, we demonstrate that the PRINT RNA template 5′ module, which as a complementary DNA 3′ end will generate the transgene 5′ junction with rDNA, influences the efficiency and mechanism of gene insertion. Iterative design and testing identified optimal 5′ modules consisting of a hepatitis delta virus–like ribozyme fold with high thermodynamic stability, suggesting that RNA template degradation from its 5′ end may limit transgene insertion efficiency. We also demonstrate that transgene 5′ junction formation can be either precise, formed by annealing the 3′ end of first-strand complementary DNA with the upstream target site, or imprecise, by end-joining, but this difference in junction formation mechanism is not a major determinant of insertion efficiency. Sequence characterization of imprecise end-joining events indicates surprisingly minimal reliance on microhomology. Our findings expand the current understanding of the role of R2 retrotransposon transcript sequence and structure, and especially the 5′ ribozyme fold, for retrotransposon mobility and RNA-templated gene synthesis in cells.

## INTRODUCTION

**FIGURE 1.**
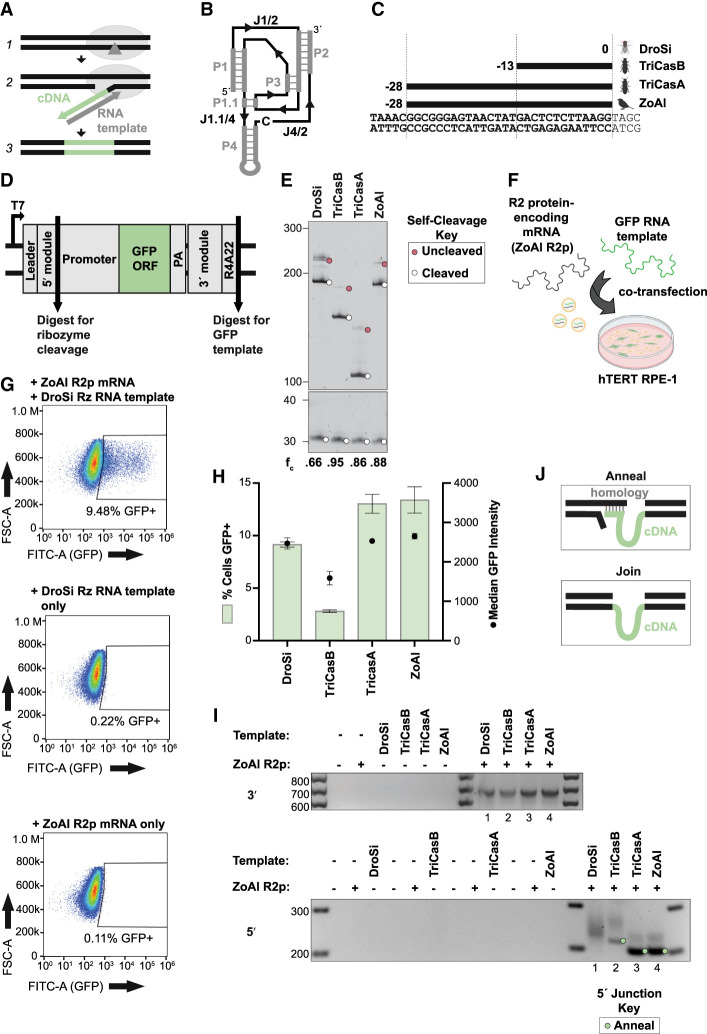
Native R2 Rz sequences confer different efficiencies of RNA template function. (*A*) Depiction of R2 retrotransposon insertion mechanism. (1) R2-encoded protein nicks first-strand DNA at insertion site. (2) R2-encoded protein generates a cDNA copy of its bound transcript by TPRT. (3) Second-strand nicking and second-strand synthesis complete R2 insertion. (*B*) HDV Rz secondary structure showing helices (gray, denoted P) and single-stranded joining regions (black, denoted J). C labels the position of the essential catalytic base. (*C*) Predicted cleavage positions (dashed vertical lines) and rRNA lengths of R2 HDV-like ribozymes aligned to the rDNA region upstream of the R2 insertion site denoted as number 0. (*D*) Plasmid construct used as a template for generation of in vitro transcription (IVT) RNAs for Rz cleavage and transgene insertion assays. (*E*) Analysis of native R2 Rz self-cleavage from a PP7 hairpin (hp) leader during a 3 h IVT reaction. Reaction products were separated by 6% Urea-PAGE and stained with SYBR Gold. Here and subsequently, uncleaved and cleaved products are indicated by red and white circles, respectively, and the fraction cleaved (*f*_*c*_) is quantified *below* each lane. (*F*) Depiction of transgene insertion assay. ZoAl R2p mRNA and green fluorescent protein (GFP) RNA template are codelivered to hTERT RPE-1 cells by lipofection. (*G*) Representative flow cytometry plots show the percentage of cells expressing GFP 1-day post-transfection of both ZoAl R2p RNA and DroSi Rz RNA template (*top*), RNA template only (*middle*), or ZoAl R2p RNA only (*bottom*). Forward scatter (FSC-A) on the *y*-axis is a proxy for cell size. (*H*) Quantification of percentage of cells expressing GFP (*left y*-axis, green bars) and median GFP intensity (*right y*-axis, black dots) as assessed by flow cytometry 1-day post-transfection of ZoAl R2p mRNA and the indicated 5′ module RNA templates. GFP% was quantified by subtracting template-only GFP% from the corresponding 2-RNA delivery GFP%. Values indicate mean ± SD, *n* = 3. (*I*) PCR amplification of 3′ (*top*) and 5′ (*bottom*) rDNA-transgene junctions from gDNA in a representative replicate of the experiment in *H*. 5′ junctions were amplified with the same rDNA-specific forward primer and different, 5′ module-specific reverse primers. Products were separated by 2% agarose gel. Here and subsequently, expected 5′ junction product bands from annealing of transgene cDNA with upstream rDNA are indicated by green circles. (*J*) Depiction of 5′ junction formation mechanisms. (*Top*) cDNA anneals to upstream rDNA. (*Bottom*) cDNA is end-joined to rDNA.

Non-long terminal repeat (non-LTR) retrotransposons are an ancient and widespread group of selfish genetic elements that mobilize within the genomes of many eukaryotes (Han 2010). The R2 family is a branch of non-LTR retrotransposons that insert site-specifically into the conserved 28S ribosomal RNA genes (rDNA) of diverse animal phyla through the mechanism of target-primed reverse transcription (TPRT) shared by non-LTR retrotransposons (Eickbush and Eickbush 2015). For R2 retrotransposons, TPRT occurs by coordinated activity of the R2 protein (R2p) N-terminal DNA-binding domains, restriction-like endonuclease (EN) domain, and reverse transcriptase (RT) domain encoded by a single open reading frame (ORF) (Eickbush and Eickbush 2015). The R2p EN makes a site-specific single-strand nick in host genomic DNA (gDNA), and its RT synthesizes complementary DNA (cDNA) by priming from the exposed 3′ OH (Luan et al. 1993). This results in cDNA insertion directly into the genome, without requirement for an integrase enzyme (Fig. 1A). The process is completed by second-strand nicking and second-strand synthesis. Biochemical and structural studies indicate that an R2p interaction with its transcript 3′ untranslated region (UTR) is essential for TPRT, whereas in some species’ R2 elements, other transcript regions may contribute to the insertion process (Luan and Eickbush 1995; Eickbush et al. 2000; Christensen et al. 2006; Deng et al. 2023; Wilkinson et al. 2023; Zhang et al. 2024).

Relatively little is understood about R2 5′ RNA regions, which have proposed roles in both primary transcript RNA processing and noncanonical translation initiation (Eickbush and Eickbush 2010; Ruminski et al. 2011). R2 retrotransposon sequences lack an identifiable promoter and are presumed to be transcribed by RNA polymerase (RNAP) I as part of an interrupted ribosomal RNA (rRNA) precursor (George and Eickbush 1999; Eickbush and Eickbush 2010). Following transcription, R2 RNA may excise from rRNA at its 5′ end by activity of a hepatitis delta virus (HDV)-like self-cleaving ribozyme (Rz), which is the only structurally defined 5′ RNA region known to be conserved across R2 phylogenetic groups (Eickbush and Eickbush 2010; Ruminski et al. 2010, 2011; Eickbush et al. 2013). The Rz fold was first characterized in HDV genomic and antigenomic RNAs but more recently has been identified in a broad diversity of genome-encoded RNA transcripts (Kuo et al. 1988; Wu et al. 1989; Webb et al. 2009).

In their native context, HDV Rzs self-cleave linear concatemeric genomic and antigenomic transcripts into monomers, which are intramolecularly ligated to generate the circular viral genome and its corresponding antigenomic template (Reid and Lazinski 2000; Macnaughton et al. 2002). HDV Rzs fold into a nested double-pseudoknot structure, which consists of five paired helices (P) linked by single-stranded joining regions (J), forming coaxial stacks (Fig. 1B; Ferré-D'Amaré et al. 1998). R2 HDV-like Rzs have the same general predicted fold but variable J1/2 regions can be long and structured (Ruminski et al. 2010). The topologically complex folding of HDV and HDV-like Rzs can be compromised by stable alternative folds, leading to only a fraction of the RNA forming a cleavage-competent structure (Chadalavada et al. 2000; Diegelman-Parente and Bevilacqua 2002). A catalytic cytosine in J4/2 is essential to efficient self-cleavage (Perrotta et al. 1999; Nakano et al. 2000). HDV Rzs are highly resistant to unfolding in vitro and have an exceptionally long half-life of ∼5 days in cultured human cells (Smith and Dinter-Gottlieb 1991; Duhamel 1996; Lévesque et al. 2002). These properties suggest that an HDV-like Rz could protect the 5′ end of a processed R2 transcript from exonucleolytic degradation.

In vitro cleavage activity of HDV-like Rzs in R2 transcripts differs widely, from near-complete self-cleavage in the fruit fly *Drosophila simulans* (DroSi R2) to little or no detectable self-cleavage in the domestic silk moth *Bombyx mori* (BoMo R2) (Eickbush et al. 2013). Some R2 Rzs self-cleave upstream of the genomic insertion site, leaving up to a predicted 36 nucleotides (nts) of residual rRNA at the 5′ end of the R2 transcript (Eickbush et al. 2013). Because inclusion of rRNA generates a cDNA 3′ end that can base-pair upstream of the target site, heteroduplex formation between the cDNA and target site could initiate 5′ junction formation and second-strand synthesis. Analyses of R2 insertions in different species indicate that 5′ junction heterogeneity, including flanking rDNA deletion events, is more frequent if Rz self-cleavage is predicted to remove all rRNA from the R2 transcript 5′ end (Eickbush et al. 2013). Insertions into rDNA of the fruit fly *Drosophila melanogaster* mediated by injection of BoMo R2p and 3′-UTR RNA yielded heterogeneous 5′ junction sequences unless the RNA template had 5′ rRNA sequence, enabling cDNA 3′-end base-pairing to the upstream target site (Eickbush et al. 2000). This contribution of RNA template 5′ rRNA could influence the efficiency of retrotransposon insertion.

To investigate the significance of R2 5′ Rz sequence and structure in the insertion process, here we compare the utility of including HDV or HDV-like Rzs or other sequences as the 5′ module of an RNA template for R2p-mediated transgene insertion to rDNA. We recently developed precise RNA-mediated insertion of transgenes (PRINT), a method that leverages avian R2 retrotransposon proteins’ remarkable specificity for RNA templates with avian R2 3′-UTR sequences to insert a transgene into rDNA (Zhang et al. 2024). The approach relies on cotransfection of an mRNA encoding R2p and an RNA template encoding a transgene, here consisting of a cassette with an RNAP II promoter, enhanced green fluorescent protein (GFP) ORF, and polyadenylation signal (PA), flanked by 5′ and 3′ modules. Gene insertions that disrupt several of the hundreds of rDNA loci per cell are well-tolerated by even rapidly dividing cells and organisms (Jakubczak et al. 1991). Our previous work examined the requirements for the PRINT RNA template 3′ module. Characterization of transgene-rDNA junctions demonstrated the formation of precise 3′ junctions resulting from accurate TPRT initiation, but heterogeneous 5′ junctions from full-length as well as 5′-truncated insertions (Zhang et al. 2024). In this study, we address how the RNA template 5′ module contributes to PRINT. Our findings indicate that an HDV or HDV-like Rz structure as the RNA template 5′ module can greatly improve transgene insertion efficiency, especially for RNA templates made using uridine (U) rather than a structure-stabilizing U analog. We also demonstrate that transgene 5′ junctions can form by annealing of short regions of cDNA–rDNA complementarity (as little as 11 bp) or by microhomology-mediated or homology-independent end-joining mechanisms, yet which mechanism is used is not the major influence on transgene insertion efficiency. These findings contribute to understanding non-LTR retrotransposon mobility and to optimizing PRINT as a molecular tool with potential genome-engineering applications.

## RESULTS

### Native R2 Rz sequences confer different efficiencies of RNA template function

R2 retrotransposons are grouped into four clades that differ in the number and type of N-terminal zinc finger domains (ZFs) preceding the DNA-binding Myb domain (Kojima et al. 2016). We developed PRINT using A-clade avian R2 proteins containing 3 N-terminal ZFs, with particular success for an R2p from the sparrow *Zonotrichia albicollis* (ZoAl R2p) (Zhang et al. 2024). In our prior work, the RNA templates paired with ZoAl R2p mRNA for codelivery to cells harbored a compact, relatively minimal length Rz 5′ module adapted from a B-clade R2 retrotransposon in the flour beetle *Tribolium castaneum* (initially annotated as R2-A, here TriCasA) (Zhang et al. 2024). To evaluate other R2 5′ Rzs as PRINT RNA template 5′ modules, we used sequences from R2 retrotransposons of different species with different predicted lengths of retained rRNA (Fig. 1C). If necessary, we adjusted the rRNA sequence retained within a cleaved Rz to match human rRNA consensus, and in some cases, the peripheral Rz P4 was stabilized with a capping stem–loop as done in structural studies (Ferré-D'Amaré et al. 1998). In vitro transcription (IVT) by T7 RNAP was used to synthesize two RNAs (Fig. 1D): one with a leader sequence and the Rz only, for testing self-cleavage, and a longer PRINT RNA template extended by the transgene expression cassette, a 3′ module for ZoAl R2p binding, and a 3′ tail with 4 nt of downstream rRNA (R4) followed by a 22-nt polyadenosine tract (A22) to promote TPRT.

We compared confirmed or predicted R2 Rzs that cleave at positions 0, −13, or −28 in upstream rRNA (Fig. 1C). TriCasA Rz previously used for PRINT RNA templates self-cleaves at position −28 (Eickbush et al. 2013). The Rz from the active D-clade DroSi R2 self-cleaves at the retrotransposon boundary with rDNA, indicated as position 0, leaving no upstream rRNA (Eickbush et al. 2013). The Rz from the A-clade R2 of the flour beetle *T. castaneum* (initially annotated R2-B, here TriCasB) self-cleaves at position −13 (Eickbush et al. 2013). We also tested the potential 5′ Rz from the A-clade ZoAl R2 that is species-matched to ZoAl R2p, predicted to cleave at position −28. RNAs synthesized to test self-cleavage extended no more than 50 nt beyond the final P2 strand of the predicted Rz (see Supplemental Fig. S1 for Rz schematics of putative secondary structure base-pairings). If Rzs are active for self-cleavage, they release a transcript 5′ leader (Fig. 1D) that is the hairpin (hp) stem–loop binding site for *Pseudomonas* phage PP7 coat protein (Lim and Peabody 2002). To evaluate self-cleavage, we resolved IVT RNA lengths by denaturing PAGE and SYBR Gold staining. All of the native-derived Rzs self-cleaved to precisely release the transcript leader with 66%–95% efficiency (Fig. 1E) under the buffer conditions of IVT RNA synthesis.

We next tested each Rz as the 5′ module of an RNA template for transgene insertion to rDNA. The RNA template transgene cassette encoded the chicken β-actin-hybrid intron (CBh) promoter, GFP ORF, and simian virus 40 (SV40) PA signal, enabling comparison of transgene insertion by flow cytometry read-out. Human telomerase reverse transcriptase (hTERT)-immortalized retinal pigment epithelial (RPE-1) cells were used for cotransfection of RNA template and ZoAl R2p mRNA (Fig. 1F). Relative transgene insertion efficiency was scored as the percentage of cells expressing GFP (GFP%) 1-day post-transfection (Fig. 1G shows representative flow cytometry data). We also compared the median GFP intensity of GFP-positive cells, which correlates with the transgene copy number inserted into the multicopy rDNA locus (Zhang et al. 2024). As negative controls, we transfected either ZoAl R2p mRNA or GFP RNA template alone. A small background of cells gated as low-intensity GFP-positive following transfection with RNA template alone (Fig. 1G), so these values were subtracted from the GFP% measured for the parallel transfection of RNA template and R2p mRNA. RNA templates with each tested 5′ module Rz supported GFP transgene insertion, resulting in GFP expression in 2%–13% of cells quantified using assays performed in triplicate (Fig. 1H).

A comparison of GFP% yielded by different R2 HDV-like Rz 5′ module RNA templates suggests several conclusions. First, the highest GFP% (∼13%) was observed with RNA templates harboring the Rzs from TriCasA used in our previous work, and ZoAl, both cleaving at rRNA position −28 (Fig. 1H; Supplemental Fig. S1). The lowest GFP% (∼2%) was observed for the RNA template with TriCasB Rz cleaving at rRNA position −13. Despite the lack of upstream rDNA homology in the cDNA produced from an RNA template with DroSi Rz 5′ module (Fig. 1C), an intermediate GFP% (∼8%–9%) was detected (Fig. 1H; Supplemental Fig. S1). Any RNA template molecules with uncleaved DroSi Rz, like RNA template with self-cleaved DroSi Rz, would lack upstream rDNA homology. We conclude that there is not an obvious relationship between an RNA template's transgene insertion efficiency and its length of 5′ rRNA.

Second, the detection of the junctions formed between rDNA and transgene, which were amplified by PCR from gDNA, gave insight about template 5′ module influence on the mechanism of 5′ junction formation. The presence of PCR products confirms insertion at the 28S rDNA locus after cotransfection of both ZoAl R2p mRNA and GFP RNA template but not either alone (Fig. 1I). No size heterogeneity was detected at the transgene 3′ junction (Fig. 1I, top), as expected from consistent TPRT initiation at the expected rDNA insertion site, congruent with previous PRINT results (Zhang et al. 2024). To detect transgene 5′ junctions, a shared primer was positioned in upstream rDNA, and different reverse primers were used for each different 5′ module. For PRINT using RNA templates with 5′ rRNA, the size of 5′ junction PCR products suggests that annealing occurred between the cDNA 3′ end and upstream rDNA (Fig. 1J, Anneal), as confirmed by sequencing (see below). Accordingly, PCR products were predominantly of homogeneous length using RNA templates with a ZoAl or TriCasA Rz 5′ module (Fig. 1I, bottom, lanes 3 and 4). In contrast, with DroSi Rz as the 5′ module, 5′ junction PCR product sizes were heterogeneous (Fig. 1I, bottom, lane 1) and their size range suggests that cDNA was end-joined to rDNA (Fig. 1J, Join). Minor transgene 5′ junction PCR products from the use of RNA templates with ZoAl or TriCasA Rz as the 5′ module were also consistent with end-joining rather than annealing (Fig. 1I, bottom, products larger than indicated by the filled circle). The 5′ junction PCR products from the use of TriCasB Rz as the RNA template 5′ module had roughly equal distribution between the length indicative of 5′ junction formation by cDNA 3′ end annealing and heterogeneous lengths consistent with end-joining (Fig. 1I, bottom, lane 2). Overall, the cross-species 5′ Rz sampling above reinforces the conclusion that efficient transgene insertion by an A-clade R2p does not require a species-matched Rz as the RNA template 5′ module. Our observations also indicate that RNA template 5′ rRNA has a direct impact on the mechanism underlying 5′ junction formation but is not strictly related to the efficiency of productive transgene insertion.

### HDV-like Rz structure contributes to RNA template 5′ module influence on transgene insertion efficiency

**FIGURE 2.**
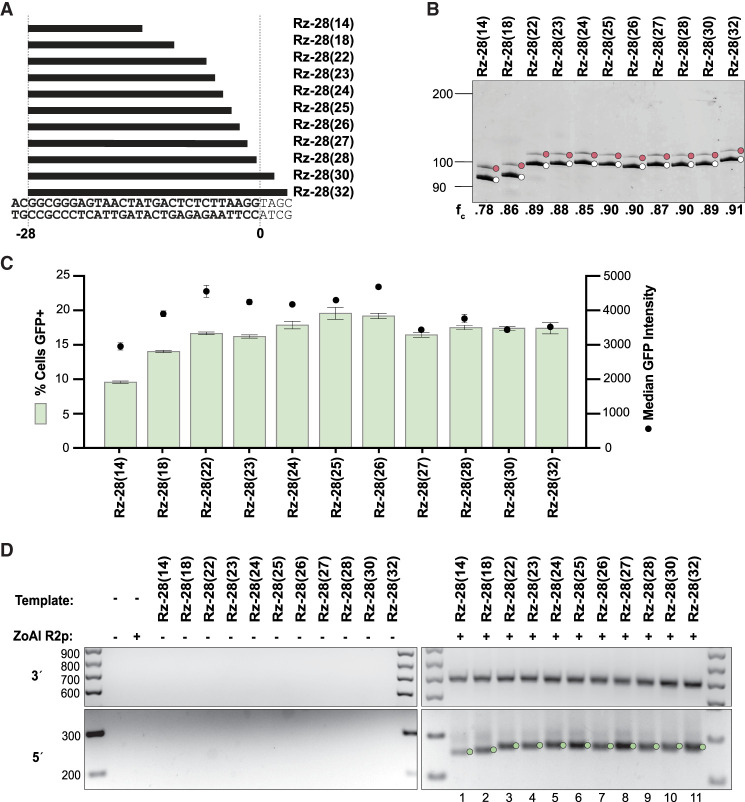
Designed HDV-like Rzs confer efficient RNA template 5′ module function. (*A*) rRNA lengths of designed Rz-28 HDV-like Rzs, aligned to upstream rDNA sequence; −28 indicates the cleavage position in the rRNA sequence, and values in parentheses indicate the number of nucleotides of rRNA sequence included. (*B*) Analysis of Rz-28 cleavage from L8 leader. Reaction products were separated by 12% Urea-PAGE. (*C*) Quantification of insertion efficiency for RNA templates with the indicated Rz-28 5′ modules as determined by flow cytometry. Values indicate mean ± SD, *n* = 3. (*D*) PCR amplification of 3′ (*top*) and 5′ (*bottom*) rDNA-transgene junctions from gDNA in a representative replicate of the experiment in *C*.

Because species-diverse R2 Rz sequences can function as a PRINT RNA template 5′ module, we next tested whether 5′ modules comprised of nonnative, designed HDV-like Rzs would support efficient transgene insertion. We designed Rzs with a P1 stem corresponding to the −28 self-cleavage position (designated Rz-28) and different lengths of rRNA sequence from −28 to 0 in the J1/2 region (Fig. 2A). Each Rz-28 version was active for self-cleavage (Fig. 2B). RNA templates with a 5′ Rz-28 module containing any length of rRNA from 22 to 32 nt, indicated as Rz-28(22) to Rz-28(32), supported comparable transgene insertion efficiency, evident in similar GFP% (Fig. 2C). Rz-28(18) and Rz-28(14) gave sequentially decreasing GFP% and lower GFP median intensity in GFP-positive cells, both indicative of fewer transgene insertions (Fig. 2C). All Rz-28 5′ module RNA templates yielded the expected precise transgene 3′ junctions and predominately precise transgene 5′ junctions (Fig. 2D).

**FIGURE 3.**
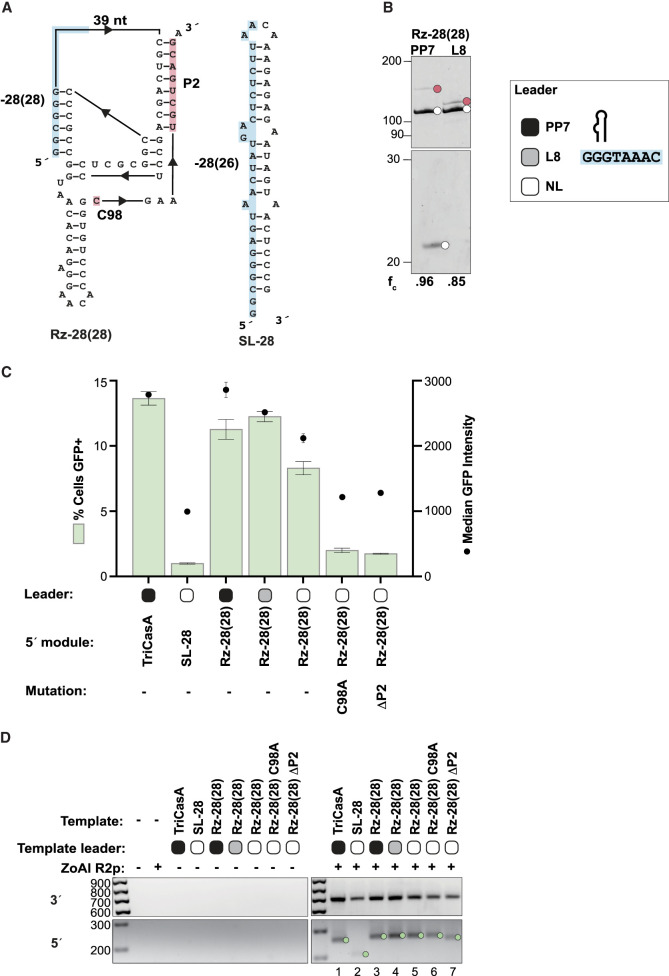
HDV-like Rz structure contributes to RNA template 5′ module function. (*A*) Predicted secondary structures of RNA template 5′ modules Rz-28(28) (*left*) and SL-28 (*right*). rRNA sequence (blue) and Rz regions mutated across the 5′ modules in this figure (pink) are indicated. (*B*, *left*) Analysis of Rz-28(28) cleavage from a PP7 hp or L8 leader sequence. Reaction products were separated by 12% Urea-PAGE. (*Right*) Depiction of the compared Rz leader sequences described in the text. (*C*) Quantification of insertion efficiency of TriCasA Rz, SL-28, and Rz-28(28) 5′ modules as determined by flow cytometry. Different Rz-28(28) leader sequences and mutated variants are indicated. Values indicate mean ± SD, *n* = 3. (*D*) PCR amplification of 3′ (*top*) and 5′ (*bottom*) rDNA-transgene junctions from gDNA in a representative replicate of the experiment in *C*.

To further investigate the role of Rz-28 in determining the efficiency of RNA template 5′ module function, we mutated various features of the Rz and its 5′-flanking leader sequence. To probe the influence of Rz structure, we disrupted or replaced features of the Rz fold (Fig. 3A). Leader sequences could impact self-cleavage, for example by the formation of alternate stable stems that impede folding of the cleavage-competent structure (Chadalavada et al. 2000; Ruminski et al. 2011). Therefore, we compared the 5′ leader PP7 hp with a leader sequence that is the 8 nt of rRNA upstream in the target site from the −28 cleavage position (L8), which were both effectively released by Rz self-cleavage in vitro (Fig. 3B). We also tested versions of Rz-28(28) that retain potential to fold as a double-nested pseudoknot but do not undergo self-cleavage: no leader (NL) Rzs with or without substitution of the catalytic cytosine (C98A) (Fig. 3A, pink-shaded C). Additionally, we made a leader-less 5′ module with Rz fold disrupted by deleting the 3′ strand of P2 (ΔP2) (Fig. 3A, pink shading). As a final comparison, we used a 5′ module consisting of a simple stem–loop with 5′-terminal 26 nt of upstream rRNA starting from the −28 position (Fig. 3A, SL-28).

PRINT GFP% with RNA templates using a 5′ module SL-28 versus an Rz, either Rz-28(28) or TriCasA Rz, indicated a clear benefit of the Rz structure (Fig. 3C, left three bars). The ΔP2 and C98A mutations were both severely inhibitory (Fig. 3C, right), whereas the Rz-28(28) NL 5′ modules gave an intermediate GFP%. All versions of the Rz-28(28) 5′ module yielded precise transgene 3′ junctions and predominately precise transgene 5′ junctions (Fig. 3D). The few insertions generated using an RNA template with the SL-28 5′ module also had predominately precise 5′ junctions expected from annealing of the cDNA 3′ end to the upstream target site (Fig. 3D, bottom). RNA templates without an active Rz would have a triphosphate 5′ end rather than the 5′ OH produced by self-cleavage, which could influence transgene insertion efficiency, for example, by inducing a RIG-I-mediated innate immune response (Pichlmair et al. 2006). We compared the SL-28 RNA template with or without phosphatase treatment and found that the presence of triphosphate did not explain the reduced efficiency of transgene insertion (Supplemental Fig. S2). Our findings best support a model in which the 5′ module Rz structure itself significantly increases the efficiency of RNA template use for transgene insertion.

### Rz cleavage position in rRNA influences 5′ module function for transgene insertion

**FIGURE 4.**
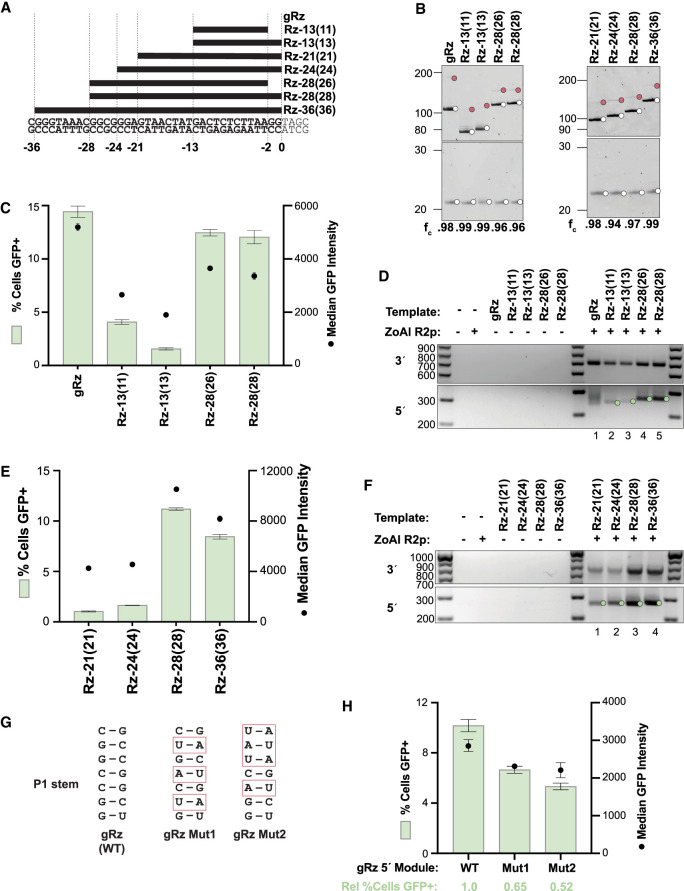
Rz cleavage position in rRNA influences 5′ module function for transgene insertion. (*A*) HDV and designed HDV-like Rz 5′ modules with variable rRNA lengths and cleavage positions within rRNA. 5′ module sequences are aligned to upstream rDNA. (*B*) Analysis of cleavage from a PP7 hp leader by HDV and designed HDV-like Rzs with variable cleavage positions. (*Left*) 5′ module Rzs relevant to *C* and *D*. (*Right*) 5′ module Rzs relevant to *E* and *F*. Reaction products were separated by 12% Urea-PAGE. (*C*) Quantification of insertion efficiency of HDV gRz and designed Rzs with −13 and −28 cleavage positions as determined by flow cytometry. Values indicate mean ± SD, *n* = 3. (*D*) PCR amplification of 3′ (*top*) and 5′ (*bottom*) rDNA-transgene junctions from gDNA in a representative replicate of the experiment in *C*. (*E*) Quantification of insertion efficiency of designed Rzs with −21, −24, −28, and −36 cleavage positions as determined by flow cytometry. Values indicate mean ± SD, *n* = 3. (*F*) PCR amplification of 3′ (*top*) and 5′ (*bottom*) rDNA-transgene junctions from gDNA in a representative replicate of the experiment in *E*. (*G*) Depiction of predicted P1 stem base-pairing for WT HDV gRz and GC-depleted mutants (Mut1 and Mut2) with substituted base pairs boxed in red. (*H*) Quantification of insertion efficiency of WT and GC-depleted mutant HDV gRzs as determined by flow cytometry. Values indicate mean ± SD, *n* = 3. Normalized fraction of cells expressing GFP relative to WT is indicated in light green.

We next queried the influence of different rRNA self-cleavage positions on the RNA template 5′ module function. To better control for sequence differences between native R2 Rzs, we used the Rz-28 scaffold to generate Rzs of minimally different sequences that self-cleave at different rRNA positions (Fig. 4A; Supplemental Fig. S3). We first compared Rz-13 and Rz-28 versions, paralleling native R2 Rzs (Fig. 1C), with rRNA lengths from the self-cleavage position to the first-strand nick (13 or 28 nt) or 2 nt shorter (11 or 26 nt), matching the 2 nt offset of first- and second-strand nicking characterized for ZoAl R2p in vitro (Luan et al. 1993; Lee et al. 2024). In parallel, we tested an RNA template 5′ module composed of the native HDV genomic ribozyme (gRz), which lacks upstream rRNA sequence (Fig. 4A; Supplemental Fig. S3). Each Rz sequence supported efficient self-cleavage in vitro (Fig. 4B). PRINT RNA templates with each Rz as a 5′ module supported transgene insertions, but Rz-13 5′ modules provided much less efficient RNA template function (∼1%–4% GFP-positive cells) than Rz-28 or gRz 5′ modules (∼11%–14% GFP-positive cells) (Fig. 4C). The rRNA position of self-cleavage was a much stronger determinant of GFP% than the 2 nt difference in rRNA length. RNA templates with any of the Rz-13 and Rz-28 5′ modules yielded the expected precise transgene 3′ junctions and predominately precise transgene 5′ junctions. The RNA template with a gRz 5′ module yielded heterogeneous transgene 5′ junctions (Fig. 4D), paralleling the 5′ junction products generated using an RNA template with a DroSi Rz 5′ module (Fig. 1I).

We expanded the panel of rRNA cleavage positions based on the HDV Rz structural requirement for a 5′ purine, which is most often a guanosine (Cerrone-Szakal et al. 2008). Designed Rz versions with −21, −24, or −36 rRNA cleavage positions (Fig. 4A) self-cleaved in vitro (Fig. 4B, right). However, PRINT RNA templates with these 5′ module Rzs showed markedly reduced transgene insertion efficiency compared to RNA templates with an Rz-28 5′ module (Fig. 4E). As expected, transgene 3′ and 5′ junctions were predominately precise (Fig. 4F). Since Rz-28 versions with a wide range of rRNA lengths support similar PRINT efficiencies (Fig. 2C), these results indicate that directly or indirectly, the Rz rRNA cleavage position greatly impacts RNA template 5′ module function. Among rRNA self-cleavage positions, Rz-28 was optimal for PRINT transgene insertions with precise 5′ junctions, whether assayed with native or designed Rzs. Additionally, we conclude that precise transgene 5′ junction formation is supported by target-site pairing with a cDNA 3′ end with as little as 11 nt of rRNA complementarity, a remarkably limited length requirement.

Differences in insertion efficiency using RNA templates with 5′ module Rzs that have different rRNA positions of self-cleavage could result from multiple factors. We noted a correlation that Rzs with a GC-rich P1 stem, such as gRz, TriCasA Rz, and versions of Rz-28, were the most effective 5′ modules for PRINT RNA templates. Comparison across HDV-like Rz P1 stems in 5′ modules of RNA templates that supported different PRINT efficiencies extended this correlation (Supplemental Fig. S4A,B). Although self-cleavage position varies across different species’ R2 HDV-like Rzs, Rz-28 is the most common (Eickbush et al. 2013), indicating that its GC-only P1 stem (Supplemental Fig. S3A) may be favorable. To investigate the influence of the GC content of the P1 stem in an Rz 5′ module independent of the rRNA sequence, we generated HDV gRz mutants with compensatory P1 base-pairing changes that decreased GC content (Fig. 4G) but still supported efficient self-cleavage (Supplemental Fig. S4C). The 5′ module gRzs with reduced P1 GC content compromised PRINT RNA template function, with the most GC-depleted variant generating ∼50% fewer GFP-positive cells that also had lower median GFP intensity (Fig. 4H). As expected, transgene insertions using RNA templates with gRZ P1 stem mutants had precise 3′ junctions and heterogeneous 5′ junctions (Supplemental Fig. S4D). These results suggest a model in which the thermodynamic stability of an HDV or HDV-like Rz fold is one determinant of an RNA template 5′ module contribution to PRINT efficiency. Because the P1 stem sequence is dependent on the position of rRNA self-cleavage, R2 Rzs that differ in the position of self-cleavage would have different levels of efficiency as the 5′ module of a PRINT RNA template.

### Uracil base modification improves transgene insertion efficiency in RNA templates with suboptimal 5′ modules

We previously reported an increase in transgene insertion efficiency using RNA templates synthesized with 100% substitution of U by pseudouridine (Ψ) or N1-methyl-pseudouridine (m1Ψ), with TriCasA Rz as the RNA template 5′ module (Zhang et al. 2024). It is understood that Ψ increases the thermodynamic stability of RNA duplexes by favoring improved base-stacking and base-pairing, which could contribute to a Ψ-mediated increase in biological stability of mRNAs (Davis 1995; Karikó et al. 2008; Hudson et al. 2013; Mauger et al. 2019). U substitution with m1Ψ is also stabilizing but appears to be more effective in suppressing innate immune responses and confers less mispairing than Ψ (Mauger et al. 2019; Morais et al. 2021). In contrast, other U analogs may negatively influence RNA stability; for example, 5-methoxy-uridine (mOU) decreases the thermodynamic stability of RNA folding (Mauger et al. 2019). We hypothesized that the influence of using U analogs in an RNA template for PRINT might vary with 5′ module RNA structure.

**FIGURE 5.**
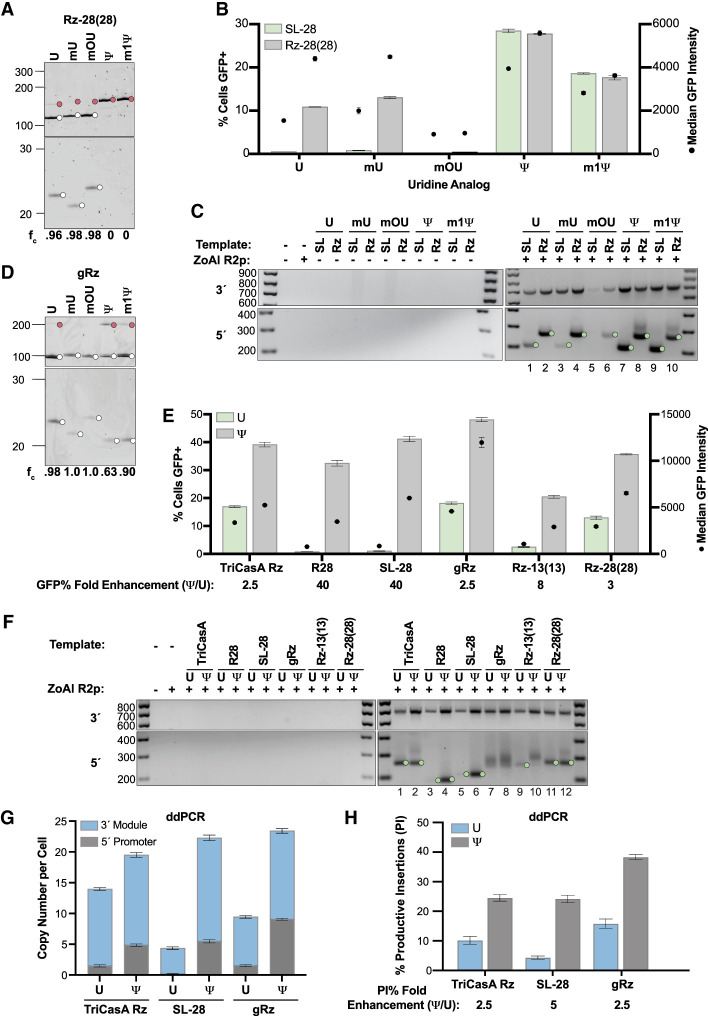
Uracil base modification improves transgene insertion efficiency in RNA templates with suboptimal 5′ modules. (*A*) Analysis of Rz-28(28) cleavage from a PP7 hp leader when either U or the substituted U analog is used for Rz synthesis. Reaction products were separated by 12% Urea-PAGE. Here and subsequently, altered mobility of the cleaved PP7 hp with different U analogs is due to folding and not differences in molecular weight (see Materials and Methods). (*B*) Quantification of insertion efficiency of SL-28 (green) and Rz-28(28) (gray) RNA templates when U or indicated U analogs are used for RNA template synthesis, as determined by flow cytometry. Values indicate mean ± SD, *n* = 3. (*C*) PCR amplification of 3′ (*top*) and 5′ (*bottom*) rDNA-transgene junctions from gDNA in a representative replicate of the experiment in *B*. (*D*) Analysis of HDV gRz cleavage from a PP7 hp leader when either U or the substituted U analog is used for Rz synthesis. Reaction products were separated by 12% Urea-PAGE. (*E*) Quantification of insertion efficiency of various 5′ module RNA templates synthesized with either U (green) or fully substituted with pseudouridine (gray), as determined by flow cytometry. Average fold enhancement, defined as the percentage of cells expressing GFP yielded by a 5′ module template synthesized with pseudouridine over the same template synthesized with U, is indicated *below* each 5′ module. Values indicate mean ± SD, *n* = 3. (*F*) PCR amplification of 3′ (*top*) and 5′ (*bottom*) rDNA-transgene junctions from gDNA in a representative replicate of the experiment in *E*. (*G*) Average transgene copy number per cell as measured within the 3′ module (blue) or 5′ promoter region (dark gray) relative to *RPP30* by ddPCR. ddPCR was performed using gDNA extracted from cells PRINTed with U or pseudouridine-substituted RNA templates. Values indicate mean ± SD, *n* = 3. (*H*) Percentage of productive insertions (PI%, the copy number of promoter relative to 3′ module) as determined from ddPCR analysis in *G*. Fold enhancement, defined as the percentage of productive insertions yielded by a 5′ module template synthesized with pseudouridine over the same template synthesized with U, is indicated *below* each 5′ module. Values indicate mean ± SD, *n* = 3.

As an initial evaluation, we compared Rz-28(28) to the simple stem–loop SL-28. Curiously, upon U replacement with 5-methyl-uridine (mU) or moU, no decrease in Rz-28(28) self-cleavage was evident (Fig. 5A, first three lanes). In contrast, upon U replacement with Ψ or m1Ψ, limited to no self-cleavage activity was detected (Fig. 5A, final two lanes). PRINT RNA templates harboring the 5′ module Rz-28(28) showed improved transgene insertion efficiency with U replacement with Ψ and to a lesser extent m1Ψ, similar insertion efficiency using mU, and severely decreased insertion efficiency using moU (Fig. 5B), paralleling results reported previously for PRINT RNA templates with TriCasA Rz as a 5′ module (Zhang et al. 2024). PRINT using an RNA template with an SL-28 5′ module showed a dramatic improvement in transgene insertion efficiency upon U replacement with Ψ, or slightly less improvement with m1Ψ (Fig. 5B). RNA templates with the Rz-28(28) versus SL-28 5′ module made using Ψ or m1Ψ supported a surprisingly similar GFP% and transgene 3′ junction PCR signal, although the Rz-28(28) RNA template generated higher median GFP intensity indicative of increased average transgene copy number (Fig. 5B,C, top). Transgene 5′ junction formation for both 5′ module RNA templates was predominantly precise (Fig. 5C, bottom). Some end-joining junction formation was detected for the Ψ- or m1Ψ-modified Rz-28(28) RNA templates (Fig. 5C, bottom), likely because these RNA templates retained non-rRNA leader sequence due to lack of Rz self-cleavage.

To extend this investigation, we created a 5′ module consisting of only 5′ rRNA (R28), with no complementary strand to form a stem–loop. As a set, we compared PRINT efficiency using RNA templates made with U or Ψ and with 5′ module R28, SL-28, gRz, Rz-13(13), Rz-28(28), or TriCasA Rz. Self-cleavage of gRz showed less compromise with any U modification (Fig. 5D), whereas the other Rzs mirrored the Rz-28(28) profile of self-cleavage inhibition by Ψ or m1Ψ (data not shown). RNA templates with the SL-28 5′ module yielded slightly higher PRINT efficiency than RNA templates with the R28 5′ module, both showing ∼40-fold increase in GFP% using Ψ versus U (Fig. 5E). The Rz-13 5′ module also supported improved transgene insertion efficiency using Ψ, with an eightfold increase in GFP%. The other 5′ module Rzs (gRz, Rz-28, and TriCasA Rz), showed a similar approximately threefold improvement using Ψ (Fig. 5E), despite the distinction that only gRZ retained efficient self-cleavage with U replacement by Ψ (Fig. 5A,D). As expected, transgene 3′ junction signal intensity paralleled GFP% (Fig. 5F, top).

Independent of synthesis with U or Ψ, most RNA templates with 5′ modules harboring rRNA generated precise transgene 5′ junctions (Fig. 5F, bottom), in contrast with the gRz 5′ module (lanes 7–8). However, of interest, with the 5′ module Rz-13, Ψ substitution entirely changed the preferred mode of transgene 5′ junction formation from annealing to end-joining, evident in longer and more heterogeneous 5′ junction PCR products (Fig. 5F, lanes 9 and 10). We propose that this altered 5′ junction formation reflects a challenge to annealing of the cDNA 3′ end with upstream rDNA when the RNA template 5′ leader sequence is retained due to Ψ substitution, which is sufficient to eliminate precise junction formation when the RNA template 5′ module contains only 13 nt of rRNA. However, retention of the RNA template 5′ leader sequence did not inhibit precise transgene 5′ junction formation to the same degree if a longer length of rRNA was present, as in RNA templates with Rz-28(28) or TriCasA Rz 5′ module. Low levels of 5′ junction end-joining PCR products were detected for RNA templates with these modules using Ψ for RNA synthesis (Fig. 5F, lanes 2 and 12).

Transgenes generated by PRINT can be 5′-truncated, reducing the number of productive insertions. Previously, we found that 5′ truncation decreased using the same RNA template with Ψ relative to U (Zhang et al. 2024). To investigate whether RNA templates with different 5′ module sequences shared a similar improvement in the percentage of intact-transgene, productive insertions (PI%) with Ψ, we used droplet digital PCR (ddPCR) to determine average transgene 5′-promoter versus 3′-end copy number relative to the reference gene *RPP30* (Zhang et al. 2024) for TriCasA Rz, SL-28, and gRz 5′ module templates synthesized with either U or Ψ. For RNA templates synthesized with U, transgene 5′-promoter and 3′-end copy number were both enhanced by the presence of a 5′ Rz (Fig. 5G, compare bar 3 with bars 1 and 5; gray bars are transgene 5′ copy number and superimposed blue bars are transgene 3′ copy number). All RNA templates synthesized with Ψ gave consistently higher copy numbers (Fig. 5G, compare bars within each set of 1–2, 3–4, and 5–6). For each RNA template, we calculated the PI% as the ratio of 5′ copy number to 3′ copy number. PI% was ∼11%, 5%, or 16% using U-containing RNA templates with TriCasA Rz, SL-28, or gRz 5′ modules, respectively (Fig. 5H). Using Ψ-containing RNA templates, PI% was ∼25%, 25%, or 39% for these same 5′ modules, respectively. The fold improvement in PI% from Ψ modification was greatest for the RNA template with the SL-28 5′ module (approximately fivefold increase; Fig. 5H). The highest PI% was achieved using the RNA template with the gRz 5′ module, whether compared in U or Ψ (Fig. 5H). Overall, we conclude that Ψ improves PRINT RNA template function for all 5′ modules, but the degree of improvement is highly dependent on 5′ module sequence and structure.

### Sequence analysis of transgene 5′ junctions suggests several mechanisms of their formation

**FIGURE 6.**
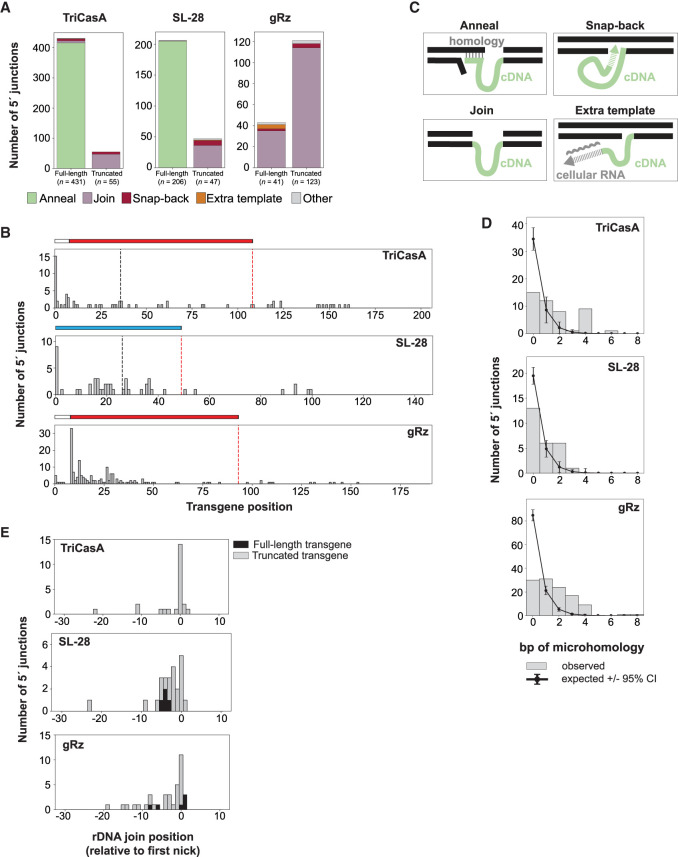
Sequence analysis of transgene 5′ junctions suggests several mechanisms of their formation. (*A*) Categorization of insertion 5′ junctions yielded by RNA templates with TriCasA, SL-28, and gRz 5′ modules. 5′ junctions are assessed as either full-length, indicating that the full amount of the nonhomology region (in the case of annealing) or total (in the case of other categories) transgene cDNA length is detected, or truncated. Within “full-length” and “truncated,” 5′ junctions are classified according to the categories in *C*. (*B*) Quantification of transgene 5′ end-joining positions for insertions yielded by different 5′ module RNA templates. Colored lines *above* templates indicate 5′ module regions, with white corresponding to the L8 leader, red corresponding to each Rz, and blue corresponding to SL-28. The remaining transgene sequence is an identical 98 nt 3′ module. Black vertical dashed line indicates the end of the region of rDNA homology. Red vertical dashed line indicates the end of 5′ module. (*C*) Depiction of different mechanisms of 5′ junction formation deduced from WGS analysis of cells with transgene insertions yielded by cotransfection of ZoAl R2p mRNA and RNA templates with different 5′ modules. (*D*) Quantification of number of nucleotides of inferred microhomology between transgene cDNA and rDNA at 5′ junctions. Nucleotides of microhomology (gray bars) are compared to the expected frequency of microhomology by chance (black lines, see Materials and Methods). (*E*) rDNA-transgene join position frequency for insertions with no detected microhomology yielded by different 5′ module RNA templates. Join positions are determined relative to the position of first-strand nicking, defined as 0. Black, full-length insertion events. Gray, truncated insertion events.

Previously, we used Illumina whole-genome sequencing (WGS) of gDNA from PRINTed cells to quantify the on-target specificity of rDNA insertion, which was >99% for ZoAl R2p (Zhang et al. 2024). Here, we turned to WGS of gDNA harvested similarly from PRINTed cells 1 day after 2-RNA delivery to query similarities and differences in the sequences of transgene junctions formed using RNA templates with or without 5′ module Rz structure and with or without rRNA homology. To maximize the number of full-length transgene insertions, RNA templates were composed of only a varying 5′ module fused to a minimized 3′ module in which GeFo 3′ UTR was truncated to its terminal 98 nt, without an internal GFP expression cassette (Supplemental Fig. S5A). RNA templates were synthesized with Ψ using TriCasA Rz, SL-28, or gRz as the 5′ module. TriCasA Rz and SL-28 share rRNA homology but differ in Rz presence, whereas TriCasA Rz and gRz share Rz structure but differ in the presence of rRNA homology. Analysis of transgene 3′ junctions indicated that cells given ZoAl R2p mRNA and any RNA template gained rDNA-inserted transgenes with very few off-target insertions (Supplemental Fig. S5B), consistent with prior analysis at greater sequencing depth (Zhang et al. 2024). Transgene 5′ junctions were classified from the transgene side as either FL or 5′-truncated (Fig. 6A). Unexpectedly, transgene insertions using an RNA template with the gRz 5′ module were often slightly 5′-truncated, most commonly within 50 nt of the 5′ terminus (Fig. 6B).

To evaluate the transgene-flanking 5′ junction sequence, we first classified four categories of junction by general mechanism of formation (Zhang et al. 2024). RNA templates with upstream rDNA homology generated 5′ junctions in the “Anneal” category, resulting from cDNA 3′ end base-pairing with upstream target site and therefore no duplication of the rDNA sequence shared by template and target site (Fig. 6C, upper left). These junctions were the predominant type for both TriCasA and SL-28 5′ modules (Fig. 6A, green bars). The “Join” category has direct fusion of cDNA to rDNA (Fig. 6C, lower left), which was the predominant type of junction for the gRz 5′ module and for 5′-truncated transgenes produced from any RNA template (Fig. 6A, purple bars). Other types of transgene 5′ junctions were observed with low frequency (Fig. 6A,C), including “Snap-back,” in which the template-complementary cDNA 3′ end is extended by priming synthesis on a nearby DNA strand before rDNA junction formation, or “Extra template,” in which the template-complementary cDNA 3′ end is extended by copying another RNA template (Zhang et al. 2024).

Using the Join category of 5′ junctions, we investigated whether the rDNA-transgene fusion occurred with potential microhomology. Although 5′ junctions occurred with potential microhomology more often than expected by chance, many junctions formed without a prediction of even 1 bp bridging the joined ends (Fig. 6D). However, some microhomology use could be obscured by ZoAl R2p-mediated nontemplated addition of one or a few nucleotides to the cDNA or upstream target-site 3′ end before junction formation (Stage and Eickbush 2009; Eickbush et al. 2013). The possibility of nontemplated sequence addition by ZoAl R2p, and/or a cellular polymerase, is supported by the detection of one or more extra nucleotides between rDNA and transgene in a minority of Join events without microhomology (Supplemental Table S1).

Also using the Join category of 5′ junctions, we defined rDNA positions of fusion to transgene sequence as a potential indication of where second-strand nicking occurs. Joins without microhomology were optimal for this analysis, because the 3′-most rDNA position could be defined unambiguously. The most common rDNA position of fusion was directly opposite the bottom-strand nick used to initiate TPRT (Fig. 6E, position 0). This precision could reflect second-strand nicking opposite the first-strand nick and/or at upstream location(s) with subsequent DNA synthesis that fills in the recessed 3′ end. Other rDNA positions of transgene joining were more heterogeneous for transgene insertions using an RNA template with the SL-28 5′ module than the TriCasA or gRz 5′ module, but they were all predominantly within 10 bp of the first-strand nick (Fig. 6E). At transgene 5′ junctions formed with potential microhomology, we determined “maximal rDNA alignment” by assigning any potential microhomology to the rDNA and “maximal transgene alignment” by assigning any potential microhomology to the transgene (Supplemental Fig. S5C–E). For these junctions as well, rDNA positions of joining to the transgene were predominantly within 10 bp of the first-strand nick. Maximal rDNA alignment recapitulated the observations for junctions with no microhomology (compare Fig. 6E; Supplemental Fig. S5E). Overall, we conclude that transgene 5′ junction formation by end-joining mechanism(s) gives similar junction features for each tested RNA template, with the major influence of the 5′ module rDNA sequence being the shift in 5′ junction formation from end-joining to cDNA 3′-end annealing with the upstream target site.

## DISCUSSION

**FIGURE 7.**
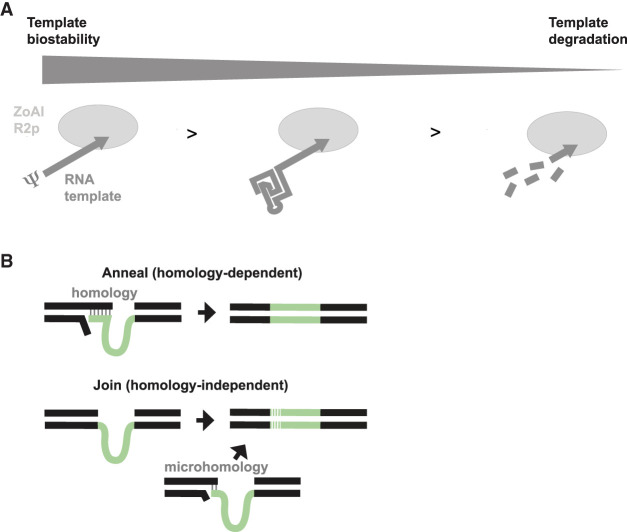
Model of RNA template 5′ module influence on PRINT efficiency and precision. (*A*) An RNA template with high biostability, imbued by a 5′ Rz structure with high thermodynamic stability or, more effectively, by synthesis with a stabilizing U analog, generates more productive insertions. RNA template instability correlates with more 5′-truncated insertions. (*B*) Separately from the 5′ module influence on RNA template biostability, the presence of 5′ homology with rRNA directs the mechanism of transgene 5′ junction formation as either Anneal or Join. Joins may occur with or without a microhomology signature.

The studies described above define several contributions of an RNA template 5′ module and its 5′-end sequence to transgene insertion by an R2 retrotransposon protein. We found that PRINT efficiency is enhanced by the presence of an HDV or HDV-like Rz at the RNA template 5′ end, especially when the Rz harbors a GC-rich P1 helix. Disruption of the Rz structure by deletion of the P2 helix greatly reduced PRINT efficiency, as did a C-to-A mutation of the essential catalytic nucleotide. A mutation at this active-site position may disrupt critical hydrogen-bonding interactions with the RNA template 5′ end (Ke et al. 2004), and the active-site substitution of a pyrimidine with a bulkier purine nucleotide is also likely to perturb Rz structure. The importance of folding stability is additionally supported by the greater influence of Rz structure when U rather than a structure-stabilizing U analog is used for RNA template synthesis. We posit that the presence of a 5′ HDV or HDV-like Rz most likely confers improved RNA template resistance to degradation (Fig. 7A) dependent on the structure but not necessarily the activity of the Rz, since Rz folding stability would be a barrier to 5′–3′ exonucleases. Indeed, Lévesque and colleagues found that the HDV Rz has an unusually long half-life in human cells, with hydrolysis patterns suggestive of primary vulnerability to endoribonuclease rather than exoribonuclease activities (Lévesque et al. 2002). Further, the 5′ OH of a self-cleaved Rz is hydrogen-bonded to the catalytic cytosine and therefore physically shielded by Rz tertiary structure (Ke et al. 2004). We suggest that the remarkable folding stability of the HDV genomic Rz, honed by selection for virus persistence, enables efficient self-cleavage even when RNA synthesis uses Ψ instead of U.

PRINT RNA template efficiency is improved by uracil base modification, with the use of Ψ rather than U in IVT RNA synthesis. A logical rationale is that use of Ψ favors more structure formation across the entire length of the RNA template and therefore more biostability when introduced to cells. However, the actual mechanism for improvement remains to be further investigated and may involve multiple factors. Ψ incorporation also reduces the innate immune response to RNA and can mitigate repression of global translation, for example, by avoiding activation of the double-stranded RNA sensor Protein Kinase R (Anderson et al. 2010). Here, we found that PRINT RNA template Ψ modification resulted in a substantial increase in productive transgene insertion as well as overall improved efficiency of insertion. As a working model, we propose that RNA template molecules can undergo 5′ truncation before transgene cDNA synthesis and that Ψ modification limits this decrease in RNA template integrity. In the future, it will be important to directly profile the integrity of TPRT-competent RNA template molecules by isolation and sequencing of R2p-bound transcripts from transfected cells.

The presence of 5′ rRNA sequence in an R2 retrotransposon or PRINT RNA template directs the mechanism by which 5′ junction formation occurs (Fig. 7B). For PRINT RNA templates, 5′ module influence on the mechanism of 5′ junction formation appears separable from its influence on insertion efficiency. With this distinction, HDV-like Rzs that retain rRNA sequence play a dual role, functioning both to confer improved transgene insertion efficiency overall and to direct the mechanism of 5′ junction formation. The transgene 5′ junctions formed with RNA templates that include 5′ rRNA sequence are implicated to form predominantly by cDNA–rDNA annealing (Fig. 7B), as evaluated both by PCR and by sequencing analysis. Without this annealing, transgene 5′ junctions appear to be formed by more than one mechanism of direct end-joining (Fig. 7B), based on the mixed presence or absence of a microhomology signature. Although many predicted R2 retrotransposon Rzs have a transcript self-cleavage position that retains 28 nt of rRNA on the R2 genomic transcript, R2 Rzs from *Drosophila* species self-cleave at the insertion site boundary (Eickbush et al. 2013). We speculate that this cleavage position may be evolutionarily favored in some situations. In *D. melanogaster*, for example, preclusion of annealing may promote rDNA unit recombination and rDNA copy number expansion, facilitating a recently described “mutualistic” relationship between retrotransposon and host (Nelson et al. 2023).

One critical remaining question is whether 5′ junction formation involves only cellular host factors or also R2p itself. Biochemical assays using BoMo R2p suggest that homology-dependent 5′ junction formation could occur by strand invasion and subsequent R2p-mediated second-strand nicking (Khadgi et al. 2019). However, based on the observation that TPRT stimulates the second-strand nicking activity of ZoAl R2p (Lee et al. 2024) and the limited length of base-pairing (11 bp) that we find sufficient for precise 5′ junction formation in this work, we favor a model in which second-strand nicking enables subsequent annealing. It remains uncertain whether R2p is involved in second-strand nicking in cells. It is also unclear whether homology-independent 5′ junction formation can be mediated by R2p template-jumping from the cDNA 3′ end to the nicked second-strand 3′ end, or vice versa, which is a possibility raised by biochemical demonstration of template-jumping for both BoMo and avian R2 proteins (Bibiłło and Eickbush 2002; Zhang et al. 2024).

Overall, our findings suggest that the biostability of the RNA template is a limiting factor for PRINT. Like PRINT RNA templates, native R2 retrotransposons may benefit from the RNA folding stability conferred by the HDV-like Rz double-pseudoknot tertiary structure. It could be critical as a substitute for the 5′ methyl-guanosine cap that later-evolved non-LTR retrotransposon transcripts possess from their gain of an internal promoter for transcription by RNAP II (Han 2010). In addition, the R2 HDV-like Rz could have other roles. HDV-like Rzs widespread in transcripts from genomes across bacteria, viruses, and animals have emerging biological roles in gene regulation and RNA processing (Webb et al. 2009; Sánchez-Luque et al. 2011; Chen et al. 2024; Kienbeck et al. 2024). Likewise, R2 HDV-like Rzs could influence rRNA processing, R2 transcript export, and/or R2p translation (Eickbush and Eickbush 2010; Ruminski et al. 2011). Although we focus here on the highly conserved R2 Rz, we note that BoMo R2p binding to a region of 5′ RNA within its coding sequence has been demonstrated to promote second-strand nicking in vitro (Christensen et al. 2006; Deng et al. 2023), suggesting that other regions of the R2 transcript 5′ end could also influence insertion through a more direct mechanism that may be species- or clade-specific. Better understanding of the full course of events required for R2 mobility, and for PRINT, is an important goal of future studies.

## MATERIALS AND METHODS

### Construct design and cloning

All plasmids were constructed using standard molecular cloning protocols. Constructs were confirmed by Primordium full-plasmid sequencing. We used the upstream rDNA sequence of the human genome, which differs in one position in *T. castaneum* rDNA. We stabilized some native P4 sequences using synthetic stem–loops adapted from binding sites for bacteriophage coat proteins (see Supplemental Fig. S1 legend). See Supplemental Table S2 for plasmids used in this study.

### Tissue culture

hTERT RPE-1 cells were obtained from the UC Berkeley Tissue Culture Facility and cultured up to 20 passages in DMEM/F12 (Gibco) supplemented with 10% fetal bovine serum (FBS) and 100 µg/mL Primocin (Invivogen). Cells were incubated at 37°C under 5% CO_2_. The cell line was validated by short tandem repeat profiling. Cells were verified to be negative for mycoplasma contamination.

### In vitro transcription and RNA preparation

N-terminally FLAG-tagged or nontagged R2 ORF mRNAs were codon-optimized and synthesized by TriLink Biotechnologies with 100% replacement of U with either mOU or m1Ψ; these yield no significant difference in PRINT efficiency (Zhang et al. 2024). Rzs and RNA templates were prepared by IVT. Plasmid templates for IVT were fully linearized by restriction digest with XbaI (NEB) for cleavage assays or BbsI-HF (NEB) for transgene insertion assays. Plasmids used to generate Rzs for cleavage assays with *Dam*-methylated XbaI restriction sites were retransformed into and isolated from *dam-*/*dcm-* competent *Escherichia coli* (NEB) before restriction digest. Linearized plasmids were column-purified using QIAquick PCR Purification kit (Qiagen) and in vitro transcribed with HiScribe T7 High Yield RNA Synthesis kit (NEB), as per the manufacturers’ instructions. Each 10 µL IVT reaction contained ∼500 ng linearized template. Where applicable, U was fully substituted for an equal quantity of modified U (TriLink or ApexBio) in the IVT reaction. Reactions were incubated at 37°C for 2 h (RNA templates) or 3 h (Rzs). Each IVT RNA was treated with 1 U DNase I (Thermo Fisher) at 37°C for 30 min. IVT products for transgene insertion assays were desalted by G25 or G50 column (IBI scientific or Cytiva), extracted with equal volume 25:24:1 v/v phenol–chloroform–isoamyl alcohol (PCI), pH 6.7 (Thermo Fisher), and precipitated with ½ volume 7.5 M LiCl overnight at −80°C. Precipitated RNA was pelleted, washed twice with ice-cold 75% ethanol (EtOH), and resuspended in 1 mM sodium citrate, pH 6.5. For RNA templates treated with calf intestinal phosphatase (CIP), 20 µg RNA was treated with 5 µL Quick CIP (NEB) at 37°C for 30 min, followed by an additional PCI extraction and LiCl precipitation step. Mock CIP treatment was performed under identical conditions using Quick CIP that had been heat-killed at 95°C for 5 min. To control for batch differences between IVT RNA, all RNA templates used in the same experiment were synthesized in parallel. RNA was assessed for quality by Urea-PAGE denaturing gel and SYBR Gold (Thermo Fisher) staining. IVT RNAs were stored at −80°C.

### Cleavage assays

Following DNase treatment, IVT RNAs synthesized in each 10 µL reaction (see above) were diluted ∼100-fold in nuclease-free water and mixed with equal volume formamide–EDTA loading buffer (95% formamide, 5 mM EDTA, 0.025% bromophenol blue, 0.025% xylene cyanol). Five microliters of the resulting solution was briefly heat-denatured and separated by 6%–12% acrylamide 7 M Urea-PAGE. Rzs were run alongside denatured DNA molecular weight ladders for size determination. Gels were stained with SYBR Gold for 15 min and imaged on an Amersham Typhoon biomolecular imager (Cytiva). Gels were quantified using ImageJ. Fraction cleaved was defined as the signal intensity of the uncleaved template over the sum of the signal intensities of the uncleaved and cleaved templates. We observed that HDV gRz and mutants had unexpected mobility patterns, likely due to some degree of folding, as migration varied with gel acrylamide concentration. We verified the uncleaved and cleaved products by comparing to a catalytically inactive control (data not shown). Synthesis with U analogs also significantly altered PP7 hp leader mobility as assessed by PAGE. Gels shown are representative of at least two replicates.

### Transgene insertion assays and flow cytometry

All transgene insertion assays were performed with hTERT RPE-1 cells that had been growing under standard conditions. Log-phase (30%–50% confluent) cells were pooled and replated at a density of 0.7–1 M cells per well of a 6-well plate. Cells were reverse-transfected with 1.5 µg total RNA, consisting of a 1:3 R2 ORF mRNA:RNA template molar ratio using Lipofectamine MessengerMAX (Thermo Fisher) in OptiMEM (Gibco), as per the manufacturer's instructions. Cells were harvested for flow cytometry by trypsinization and resuspension in either reduced serum (5% FBS) DMEM/F12 (Gibco) or DPBS (Gibco) containing 0.5 mM EDTA and 2% FBS. Cells were harvested 20–24 h post–transfection. All flow cytometry experiments were carried out on an Attune NxT Flow Cytometer (Thermo Fisher) with the voltage settings FSC 70 V, SSC 280 V, BL1 (GFP) 250 V. Data analysis was performed in FlowJo v10. Transgene insertion assays were carried out at least twice and relative transgene insertion efficiencies were reproducible between biological replicates. Following flow cytometry, equivalent volumes of cells were pelleted, washed once with DPBS (Gibco), snap-frozen in liquid N_2_, and stored at −80°C before downstream analysis.

### Junction genotyping

5′ and 3′ transgene insertion junctions were analyzed by genotyping PCR on samples that had been analyzed by flow cytometry. Frozen cell pellets were thawed on ice, resuspended in quick lysis buffer (20 mM HEPES, pH 8; 2 mM MgCl_2_; 0.2 mM EGTA; 10% glycerol; 50 mM NaCl; 1% TX-100), and digested with Proteinase K (Thermo Fisher) at 50°C for 3 h. Proteinase K was heat-inactivated at 95°C for 5 min, and 2 µL lysate was used as a template for PCR. All genotyping PCRs were carried out using Q5 DNA polymerase (NEB), as per the manufacturer's instructions. Native R2 Rz 5′ junctions (Fig. 1I) were amplified using the forward primer Geno 28S 5′ FW2 and Rz-specific reverse primers (see Table 1). All other 5′ junctions were amplified using the forward primer Geno 28S 5′ FW2 and the reverse primer Geno Term RV. All 3′ junctions were amplified with either the forward primer Geno GFP FW1, Geno GFP FW2, or Geno GFP FW3 and the reverse primer Geno 28S 3′ RV1. Thermocycler conditions were as follows: (98°C, 3 min) × 1; (98°C, 10 s; 65°C, 30 s; 72°C, 15 s) × 5 with annealing temperature decreasing by 1°C per cycle; (98°C, 15 s; 60°C, 30 s; 72°C, 15s) × 25; (72°C, 20 s) × 1. All PCR products were run on 2% agarose gels containing ethidium bromide and imaged using the BioRad GelDoc XR + imaging system. Gels shown are representative of at least two replicates.

**TABLE 1.** PCR primers

Primer name	Figure used	Sequence
Geno 28S 5′ FW2	1I, 2D, 3D, 4D, 4F, 5C, 5F, S4D	5′-CCAGGGGAATCCGACTGTTTAATTAAAACAAAGC-3′
DrSi RV	1I	5′-CCTTCCGCACACCATATGATATGACTTACGAATTAC-3′
TriCasB RV	1I	5′-GCATAACTTTTCAAGTGTGATCTGATCAGGATCAC-3′
TriCasA RV	1I	5′-CCCTGTGCCCAGCGGGAC-3′
ZoAl RV	1I	5′-GCGATCTCTGCAGCTATGCCCAG-3′
Geno Term RV	2D, 3D, 4D, 4F, 5C, 5F, S4D	5′-CTAGAAGGTCGACCAGATGTCCGAGGTCG-3′
Geno GFP FW1	1I	5′-CACTCTCGGCATGGACGAGCTGTAC-3′
Geno GFP FW2	2D, 3D, 4D, 5C, 5F	5′- CGATCACATGGTCCTGCTGGAGTTC-3′
Geno GFP FW3	4F, S4D	5′-CCCTGAGCAAAGACCCCAACGAGAAG-3′
Geno 28S 3′ RV1	1I, 2D, 3D, 4D, 4F, 5C, 5F, S4D	5′-CCACTTATTCTACACCTCTCATGTCTCTTCACC-3′

### Droplet digital PCR

ddPCR analysis was carried out on gDNA isolated from hTERT RPE-1 cells that had been analyzed by flow cytometry. Frozen cell pellets were thawed on ice and resuspended in RIPA lysis buffer (150 mM NaCl; 50 mM Tris–HCl, pH 7.5; 1 mM EDTA; 1% TX-100; 0.5% sodium deoxycholate; and 0.1% SDS; 1 mM DTT). Lysate was digested with RNase A (Thermo Fisher) at 37°C for 30 min, followed by Proteinase K (Thermo Fisher) at 50°C overnight. gDNA was isolated by extraction with equal volume PCI and precipitation with 0.1 volume 5 M NaCl and 3 volumes 100% EtOH for at least 2 h at −20°C or briefly in liquid N_2_. gDNA was pelleted, washed twice with ice-cold 75% EtOH, and resuspended in nuclease-free water. ddPCR was carried out essentially as previously described (Zhang et al. 2024) using the primers and probes listed in Table 2. *RPP30*, which we found previously to have a copy number of 3 in hTERT RPE-1 cells, was used as the reference gene for copy number analysis (Zhang et al. 2024). We found here that probes designed for the CBh promoter region detected a copy number of 1 in negative control samples from parental hTERT RPE-1 cells due to an identical promoter region within the plasmid used for cell line immortalization (Bodnar et al. 1998), and we subtracted this value as background.

**TABLE 2.** ddPCR primers/probes

Primer name	Sequence
CBh FW (oJM358)	5′-ACGCCAATAGGGACTTTCCAT-3′
CBh RV (oJM359)	5′-ACGTCAATAGGGGGCGTACT-3′
GeFo3 FW (oJM351)	5'-CCGGACTTGTCATGATCTCC-3'
GeFo3 RV (oJM285)	5'-CCGGGTTAAGTAAAGGTGGC-3'
RPP30 FW (oJM366)	5'-GATTTGGACCTGCGAGCG-3'
RPP30 RV (oJM367)	5'-GCGGCTGTCTCCACAAGT-3'

Probe name	Sequence
CBh (prJM005)	5′-/56-FAM/ACGGTAAAC/ZEN/TGCCCACTTGGCAGTACA/3IABkFQ/-3′
GeFo3 (prJM001)	5'-/56-FAM/CCGTCCACC/ZEN/TTGGACAAGTCTG/3IABkFQ/-3'
RPP30 (prJM011)	5'-/5HEX/CTGACCTGA/ZEN/AGGCTCTGCGCG/3IABkFQ/-3'

### Whole-genome sequencing

hTERT RPE-1 cells were transfected using the method described above. Twenty-four hours post-transfection, cells were harvested. gDNA was isolated using the method described above for ddPCR, except that an additional PCI extraction step was performed and gDNA was resuspended in Tris-EDTA (TE) buffer. Insertion at the 28S locus was confirmed by PCR genotyping, and gDNA quality was verified by agarose gel before sample submission. gDNA samples were submitted to the Vincent J. Coates Genomics Sequencing Lab at UC Berkeley for 30× coverage whole-genome shotgun sequencing. Briefly, gDNA was sheared to 400–500 bp with Covaris tubes for Illumina library preparation. PE150 sequencing was performed on a NovaSeq 6000 instrument with an S4 flow cell. Bioinformatic analyses were performed on the Berkeley Research Computing Savio cluster with SLURM job scheduling or on an Apple M1 Max processor.

Bioinformatic analyses were performed largely as described previously (Zhang et al. 2024). PCR and optical duplicates were removed with BBMap v38.97 (https://sourceforge.net/projects/bbmap/), and reads were trimmed for quality with Trimmomatic v0.39 (Bolger et al. 2014). Reads <36 bp in length or with a PHRED quality <30 were discarded. Alignments were performed with bwa mem v0.7.17 with default parameters (Li 2013). Reads were first aligned to a transgene reference with 840 bp of flanking rDNA on each side of the insertion. Unmapped mate pairs and clipped portions of reads were then mapped to a consensus rDNA scaffold (GenBank KY962518.1). Read mates and clipped portions of reads remaining unaligned were then mapped to the human genome reference (T2T-CHM13v2.0) (Nurk et al. 2022). Finally, still-unaligned mates and clipped reads were checked for alignment to the context surrounding the 28S insertion site with regular expression (regex; using Python fuzzysearch; https://github.com/taleinat/fuzzysearch). Reads without both mates mapped or reads aligning better to the human genome than to the transgene were discarded. Reads were also mapped to a curated list of contaminants arising from pooled sequencing; those mapping to contaminants were discarded.

3′ transgene junctions were used to classify transgene insertions as “on-target,” “rDNA off-target,” or “genomic off-target” if the insertion began within ±3 bp of the known 28S target site, elsewhere within rDNA, or anywhere else in the human genome, respectively.

5′ junctions were classified as “anneal,” “join,” “snap-back,” “extra template,” or “other.” Anneal junctions were classified as those consistent with annealing of homologous cDNA and upstream target-site sequence, producing a seamless insertion of the transgene sequence. Joined junctions were identified by a direct fusion of transgene sequence to upstream rDNA, sometimes containing additional unmapped sequence in between the rDNA and transgene sequences. A catalog of these unmapped sequence insertions for the gRz WGS sample is included in Supplemental Table S1. Snap-back junctions are characterized by fusion of reverse-complemented sequence from the transgene cDNA or nearby rDNA consistent with snap-back synthesis. 5′ junctions with upstream sequence aligned elsewhere in the human genome were categorized as “extra template” or “other.” Those consistent with copying of a second RNA template were classified as “extra template,” and all others were classified as “other.”

Maximum possible microhomology at 5′ sequence junctions was inferred by counting how many bases at the junction could map identically to either the upstream or downstream reference sequence. The expected number of junctions with *x* bp of microhomology was estimated as: $N\times \left(\frac{3}{4}\right)\times {\left(\frac{1}{4}\right)}^{x}$, where *N* is the total number of join junctions analyzed for microhomology in each data set. To estimate 95% confidence intervals, *N* observations were sampled randomly from a binomial distribution with *p* = 0.75. This sampling procedure was repeated 100,000 times. The standard deviation for the number of times *x* bp of microhomology occurred by chance across 100,000 samples was calculated and the error bars show twice the standard deviation above and below the expected value.

Python and bash scripts to reproduce the analyses in this paper from raw data are available on GitHub: https://github.com/collinslab-berkeley/R2_transgene_analysis.

## SUPPLEMENTAL MATERIAL

Supplemental material is available for this article.

## COMPETING INTEREST STATEMENT

S.M.P., C.A.H., X.Z., and K.C. are listed inventors on patent applications filed by the University of California, Berkeley related to PRINT. X.Z. and K.C. have equity options in Addition Therapeutics, Inc., which licensed the UC Berkeley technology.

## Figures and Tables

**Sarah M. Palm:**
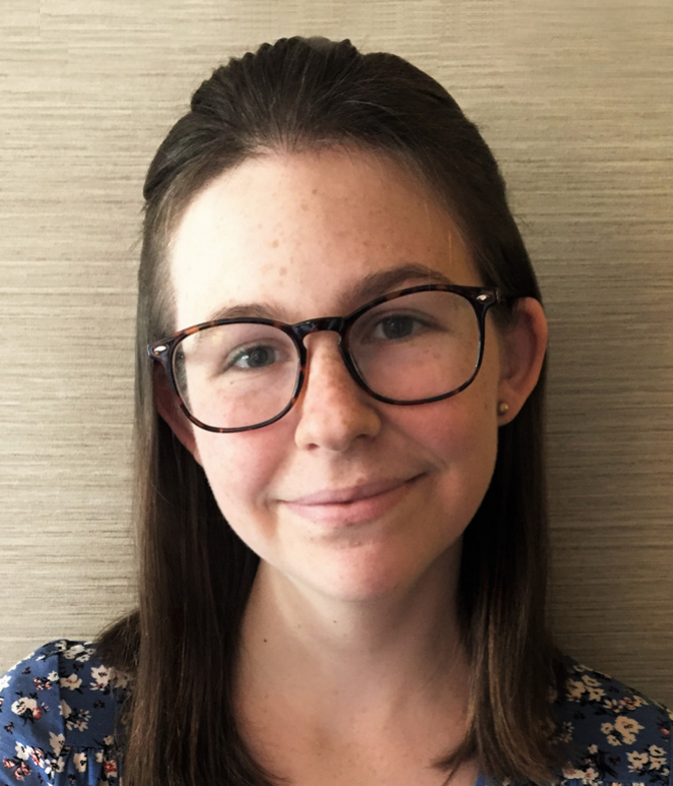


## MEET THE FIRST AUTHOR

***Meet the First Author(s)* is an editorial feature within *RNA*, in which the first author(s) of research-based papers in each issue have the opportunity to introduce themselves and their work to readers of *RNA* and the RNA research community. Sarah M. Palm is the first author of the paper, “Structure and sequence at an RNA template 5′ end influence insertion of transgenes by an R2 retrotransposon protein.” Sarah completed her bachelor's degree in Biochemistry at Scripps College and is now a PhD student in the Molecular and Cell Biology program at UC Berkeley, where her research in Professor Kathleen Collins’ lab focuses on the biology of eukaryotic retrotransposons.**

**What are the major results described in your paper and how do they impact this branch of the field?**

Previously, we developed an R2 retrotransposon-based genome-engineering method termed precise RNA-mediated insertion of transgenes (PRINT), in which transgene addition to the human genome is achieved solely by the delivery of two in vitro-transcribed RNAs. The first is an mRNA encoding the R2 protein, and the second is an RNA template that is recognized by the R2 protein and efficiently reverse-transcribed directly into the genome by target-primed reverse transcription (TPRT). This work describes 5′ features of endogenous R2 transcripts which, when included at the PRINT RNA template 5′ end, contribute to both the efficiency and precision of PRINT. First, we found that the addition of a hepatitis delta virus–like ribozyme to the 5′ end of the RNA template significantly increases both transgene insertion efficiency and the number of inserted full-length transgenes, suggesting that this RNA fold contributes to RNA template integrity. Second, we found that the inclusion of 5′ sequence homology with the region upstream of the R2 target site within 28S rDNA generates precise 5′ junctions formed by annealing with 3′ cDNA. In the lack of 5′ sequence homology, 5′ junctions are imprecise and are enriched for but do not necessarily include a microhomology signature. Our work lends insight into the mechanism(s) of retrotransposon mobility and how to most effectively leverage R2 retrotransposon transcript RNA signatures for R2 protein–mediated delivery of a transgene payload.

**What led you to study RNA or this aspect of RNA science?**

Personally, I am fascinated by the multifunctional role of retrotransposon RNA in facilitating the successful propagation of these selfish mobile genetic elements, with features contributing to RNA processing, protein translation, RNP formation, and TPRT. I am also motivated by the daily excitement of Collins Lab members as they seek to understand the unique biology of R2 retrotransposons, spanning from their biochemical properties and life cycle to their biotechnology applications.

**During the course of these experiments, were there any surprising results or particular difficulties that altered your thinking and subsequent focus?**

One surprising finding of this study was that many imprecise 5′ junctions are formed in the absence of microhomology. Investigation of the pathways underlying 5′ junction formation, including delineating the roles of the R2 protein from cellular host factors that may be involved, will be critical to our understanding of and manipulation of PRINT as a genome-engineering tool.

**Are there specific individuals or groups who have influenced your philosophy or approach to science?**

I am grateful to my mentors at U.C. Berkeley for fostering an incredible environment in which to think about and do science. My first laboratory experience at Berkeley with Professor Jeremy Thorner piqued my interest in the dynamic nature of cell signaling and its intersection with RNA biology, which inspired me to pursue a PhD. Likewise, Professor Kathleen Collins’ deep investment in her trainees and their projects has greatly increased my learning and growth as a scientist.
